# Digital Health Tool to Assist Older Adults in Self-Disclosing Elder Mistreatment

**DOI:** 10.1093/geroni/igaa057.2724

**Published:** 2020-12-16

**Authors:** Fuad Abujarad, Esther Choo, Michael Pantalon, Karen Jubanyik, James Dziura, Chelsea Edwards, Gail D’Onofrio, Thomas Gill

**Affiliations:** 1 Yale University, New Haven, Connecticut, United States; 2 Oregon Health & Science University, Portland, Oregon, United States; 3 Yale University School of Medicine, New Haven, Connecticut, United States

## Abstract

Elder Mistreatment (EM) is a global health issue that continues to be under detected and inadequately addressed in healthcare settings. In this symposium, we will describe how we built and currently use the VOICES digital health tool to screen for EM. The tool is designed exclusively with older adults in mind and runs on iPads to be used in the emergency department. VOICES screens, educates, uses motivational interviewing to facilitate self-disclosure of EM, and refers patients for in-person screening. The tool includes multimedia components such as videos, audio and animations designed to educate and enhance screening for EM. Patients who screen positive are then guided through an automated Brief Negotiated Interview (BNI) that uses motivational interviewing to help them self-identify and self-disclose. Our tool will help identify cases of EM that otherwise may go undetected and overcomes major challenges associated with traditional methods of screening.

